# Monitoring Cigarette Smoking and Relapse in Young Adults With and Without Remote Biochemical Verification: Randomized Brief Cessation Study

**DOI:** 10.2196/47662

**Published:** 2023-07-27

**Authors:** Erin A McClure, Nathaniel Baker, Kyle J Walters, Rachel L Tomko, Matthew J Carpenter, Elizabeth Bradley, Lindsay M Squeglia, Kevin M Gray

**Affiliations:** 1 Department of Psychiatry and Behavioral Sciences Medical University of South Carolina Charleston, SC United States; 2 Hollings Cancer Center Medical University of South Carolina Charleston, SC United States; 3 Department of Public Health Sciences Medical University of South Carolina Charleston, SC United States

**Keywords:** technology, mHealth, young adults, cessation, relapse, biochemical verification, cigarette, smoking, monitoring, abstinence, mobile phone

## Abstract

**Background:**

Technological advancements to study young adult smoking, relapse, and to deliver interventions remotely offer conceptual appeal, but the incorporation of technological enhancement must demonstrate benefit over traditional methods without adversely affecting outcomes. Further, integrating remote biochemical verification of smoking and abstinence may yield value in the confirmation of self-reported smoking, in addition to ecologically valid, real-time assessments.

**Objective:**

The goal of this study was to evaluate the impact of remote biochemical verification on 24-hour self-reported smoking and biochemical verification agreement, retention, compliance with remote sessions, and abstinence during a brief, 5-week cessation attempt and relapse monitoring phase.

**Methods:**

Participants (N=39; aged 18-25 years; mean age 21.6, SD 2.1 years; n=22, 56% male; n=29, 74% White) who smoked cigarettes daily engaged in a 5-week cessation and monitoring study (including a 48-hour quit attempt and provision of tobacco treatment in the form of nicotine replacement therapy, brief cessation counseling, and financial incentives for abstinence during the 2-day quit attempt only). Smoking (cigarettes per day) was self-reported through ecological momentary assessment (EMA) procedures, and participants were randomized to either (1) the inclusion of remote biochemical verification (EMA + remote carbon monoxide [rCO]) 2× per day or (2) in-person, weekly CO (wCO). Groups were compared on the following outcomes: (1) agreement in self-reported smoking and breath carbon monoxide (CO) at common study time points, (2) EMA session compliance, (3) retention in study procedures, and (4) abstinence from smoking during the 2-day quit attempt and at the end of the 5-week study.

**Results:**

No significant differences were demonstrated between the rCO group and the wCO (weekly in-person study visit) group on agreement between 24-hour self-reported smoking and breath CO (moderate to poor), compliance with remote sessions, or retention, though these outcomes numerically favored the wCO group. Abstinence was numerically higher in the wCO group after the 2-day quit attempt and significantly different at the end of treatment (day 35), favoring the wCO group.

**Conclusions:**

Though study results should be interpreted with caution given the small sample size, findings suggest that the inclusion of rCO breath added to EMA compared to EMA with weekly, in-person CO collection in young adults did not yield benefit and may have even adversely affected outcomes. Our results suggest that technological advancements may improve data accuracy through objective measurement but may also introduce barriers and burdens and could result in higher rates of missing data. The inclusion of technology to inform smoking cessation research and intervention delivery among young adults should consider (1) the research question and necessity of biochemical verification and then (2) how to seamlessly incorporate monitoring into personalized and dynamic systems to avoid the added burden and detrimental effects to compliance and honesty in self-report.

## Introduction

Cigarette smoking remains the leading cause of preventable death and disease in the United States [1-3]. Cigarette smoking among adolescents (aged 12-17 years) and young adultd (ages 18-25 years) continues to decline [4,5]; however, estimates show that 6%-9% of high school students in the United States report smoking cigarettes [4,6-8], while smoking among young adults is estimated to be between 8% and 14% [5,9]. Previous estimates suggest that the majority of adult smokers start prior to the age of 18 years [2,10], though recent studies show that smoking onset is higher in young adulthood than in adolescence [11]. Young adults who smoke cigarettes show interest in quitting and have higher odds of recent quit attempts compared to those 25 years of age or older [12-14], with some evidence of equivalent or higher cessation rates compared to older age groups [13,14]. Even so, quit attempts rarely result in successful abstinence for young adults [15-17], even when evidence-based treatments are used [18-20], and young adults remain a critical population for smoking cessation interventions.

Evaluation of novel treatment strategies among young adults is limited by traditional study designs and methods of data collection. The accuracy and veracity of self-reported smoking as a primary end point have been problematic, and inaccuracies have been attributed to factors, such as forgetting, dishonesty, underreporting, response fatigue, or disengagement in procedures [21-26]. Further, high rates of study attrition and missing data compromise power and the ability to detect differences in intervention arms [20,27-29]. Particularly among youth, the incorporation of technology may improve the accuracy of data collection as well as increase engagement and retention. Indeed, literature has documented appeal, benefit, and overall acceptance of telemedicine practices among adolescents and young adults [30,31].

Ambulatory assessment of smoking may contribute to more efficacious interventions and improved outcomes. Ecological momentary assessment (EMA) [32] samples smoking patterns in one’s natural environment, inclusive of information on context, affect, mood, stress, and other relevant variables that may be associated with or precede substance use. EMA has been used to better understand adolescent and young adult smoking, lapse, relapse and contextual, mood, and other relevant factors [26,33-36]. There may be value in integrating biochemical verification to confirm self-reported smoking, in addition to ecologically valid, real-time assessments via EMA [22,37] while still retaining methodological rigor [38]. EMA paired with remote biochemical verification may improve data accuracy and reduce the likelihood of misreporting. In addition, the capture of biochemical verification through monitors or other novel devices that quantify behavior may increase engagement and improve compliance. Remote biochemical verification also reduces the need for in-person visits, potentially increasing retention.

While technological advancements to study young adult smoking offer conceptual appeal, incorporation of these methods must demonstrate benefits over traditional methods without adversely impacting outcomes (ie, compliance, retention, and response fatigue). In a single-arm, proof-of-concept, feasibility study by our group, we implemented remote biochemical verification, in addition to self-report during a brief quit attempt and monitoring period (11 days) among young adults. We found that cigarette entries decreased across the 11 days (a mix of low rates of smoking and days of no cigarette entries), and missed sessions also increased. Given the inclusion of frequent, remote biochemical verification, we suspect that smoking was likely being underreported [22]. That study illustrated the importance of objective, frequent biochemical verification to accurately monitor smoking during and following a quit attempt, but the added benefit is unknown. The purpose of this study was to evaluate the benefit of technological enhancement among young adults who smoke cigarettes daily engaging in a brief cessation study. All participants reported their smoking through real-time cigarette entries logged on a smartphone app and were randomized to either (1) the inclusion of twice daily remote biochemical verification (carbon monoxide [CO]) integrated through the app (EMA + remote CO rCO) and weekly phone visits or (2) weekly, in-person study visits to collect CO (EMA + weekly CO wCO). The remote CO (rCO) group was compared to weekly (wCO) controls on (1) agreement in past 24-hour self-reported smoking and biochemical verification at common study time points, (2) retention in study procedures during the 5-week study, (3) compliance with EMA sessions, and (4) smoking abstinence.

## Methods

### Participants

Young adults who smoked cigarettes daily were recruited from the Charleston, South Carolina, community from April 2017 through May 2019 through social media advertisements, fliers, word of mouth, friend referrals, etc. Participants had to meet the following inclusion criteria: (1) being 18-25 years old, (2) smoking at least 5 cigarettes daily for at least 3 months, (3) willing to limit or abstain from other tobacco products, and if applicable, limit or abstain from using cannabis during the study, (4) willing to engage in a 48-hour quit attempt, and (5) having interest in quitting smoking (defined as 5 or above on a 10-point scale). While young adults who vaped nicotine (e-cigarettes) primarily were allowed into the study in the final months of enrollment, these participants (n=7) were removed from this analysis given that CO cannot be used to verify nicotine vaping. Exclusion criteria included (1) any serious or unstable psychiatric or medical disorder or any other concern that may impact safety, compliance, or could confound results, (2) current use of smoking cessation medication, or (3) pregnancy or plans to become pregnant in the next month. There were no exclusions for smartphone access, and participants were loaned a study smartphone, if they did not have one. Results from this study focused on emotion differentiation, and using EMA data affect and craving have been published elsewhere [39].

**Figure 1:**
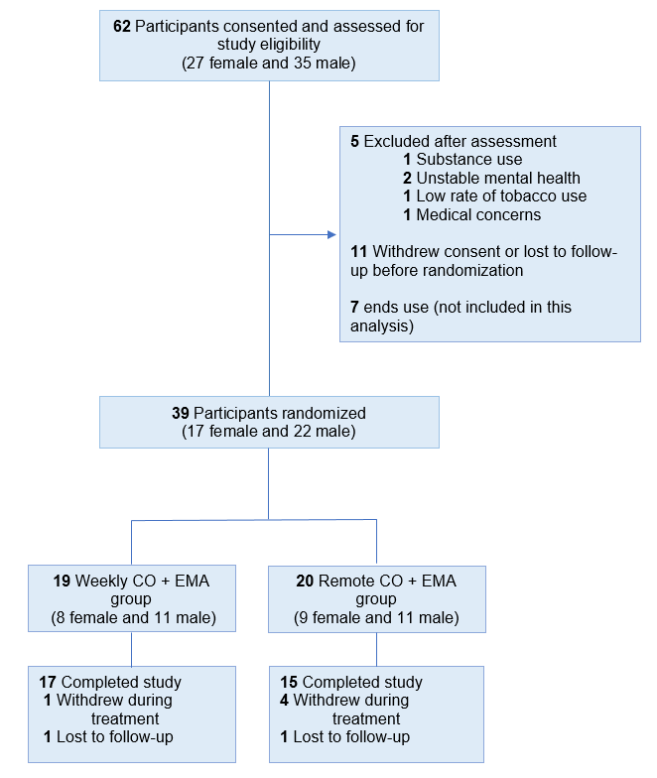
CONSORT (Consolidated Standards of Reporting Trials) diagram. CO: carbon monoxide; EMA: ecological momentary assessment.

A total of 62 participants were screened (Figure 1), 39 of whom met full criteria and were randomized 1:1 into the in-person, wCO group (n=19) or the rCO group (n=20). A total of 7 participants withdrew consent (n=5) or were lost to follow-up (n=2) during the study (n=32 study completers).

### Study Procedures

#### Study Design

**Figure 2:**
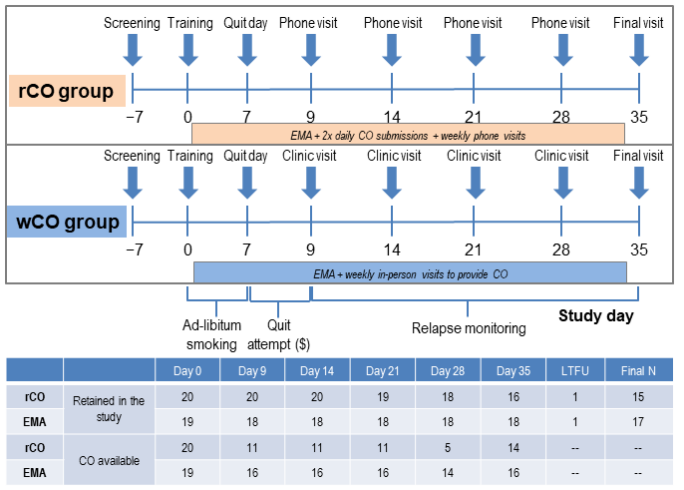
Study design figure demonstrating procedural differences between the rCO and wCO groups as well as the number of participants retained throughout the study at common time points and those with CO samples available at those time points. A participant may have missed a study visit even though they were still engaged in the study. The rCO group was asked to complete 2× daily CO samples starting on day 1 until day 34 + EMA sessions, in addition to weekly study phone visits. The wCO group was asked to complete EMA sessions from days 1 to 34 and weekly in-person visits, in which CO was obtained 1× per week. $ = abstinence incentives in US dollars were provided during the quit attempt based on the submission of negative CO samples (US $20 total per group for abstinence). Tobacco treatment was provided to all participants at day 0 and included a 2-week starter pack of nicotine replacement therapy (patches and lozenges), as well as brief cessation counseling. CO: carbon monoxide; EMA: ecological momentary assessment; LTFU: lost to follow-up; rCO: remote CO; wCO: weekly CO.

The study design is shown in Figure 2, which highlights the differences between groups. Study conditions were designed to be similar and varied on the method of CO collection (remote vs in-person), frequency of CO (weekly vs 2× per day), and conduct of weekly study visits (remote vs in-person). Randomization to either the rCO group (EMA + remote CO collection [rCO]) or the wCO group (EMA + weekly in-person visits and wCO collection) was balanced on gender and severity of smoking (cigarettes per day at screening). At the randomization visit (day 0), all participants downloaded the monitoring app (EMA-enabled app with or without rCO collection integrated; My Mobile Monitor group) onto their mobile phones and were asked to log all cigarettes smoked and complete EMA sessions for 5 weeks. Those in the rCO group were trained on the use of breath CO monitors to use remotely and the submission of CO videos. All participants were asked to make a 48-hour quit attempt starting on the morning of day 7. The relapse monitoring period began on day 9 and lasted through the end of the study (day 35). Participants in the rCO group completed weekly study visits over the phone and returned for a final in-person visit at day 35, whereas participants in the wCO group attended weekly in-person visits to submit breath CO samples.

#### Quit Attempt and Treatment Provided

The quit attempt began on day 7 and lasted through day 9 (48 hours), though it may have lasted longer per participant discretion. All participants were provided with a 2-week starter pack of nicotine replacement therapy (NRT; transdermal patches and nicotine lozenges [14 or 21 mg patches and 4 mg lozenges]) at day 0, brief cessation counseling from study research staff leading up to their quit attempt (screening and day 0: ~5-10 minutes per study visit), and financial incentives for abstinence only during the quit attempt (days 7-8). Financial incentives for abstinence ceased after day 9. Treatment delivery was identical across groups, with the exception of incentive delivery. During the quit attempt (days 7-8), incentives were provided to promote abstinence for both groups (US $40 possible). Abstinence was verified for the rCO group by remote breath CO submissions through the app (2 submissions each day on days 7 and 8 to confirm abstinence). For the wCO group, abstinence was verified by a single in-person CO sample collected at the day 9 visit. Abstinence was defined as ≤6 parts per million (ppm) or a CO value with a 75% reduction from baseline smoking CO averages. The CO cutoff of 6 ppm was based on recommendations in the literature [40], but to account for high ad-lib smoking levels prior to the quit attempt, a percent reduction cut-off was also used to not discourage abstinence among those smoking more heavily. Given the inclusion of rCO for 1 group, abstinence could be determined over several time points versus the single time point obtained for the in-person wCO group.

#### Remote Monitoring

Participants in both groups were asked to complete EMA sessions (up to 4 prompts per day plus an evening report) as well as log cigarettes smoked in the app in real time. Days consisted of participant-selected 12-hour blocks within which sessions were prompted. Two event-based EMA sessions were prompted each day following cigarette entries. Random EMA session prompts were delivered throughout the day for at least 30 minutes following the last cigarette entry. EMA items were repeated, and CO samples were required for the rCO group (video submission integrated through the app, 2 samples expected or prompted each day). Two CO samples per day were deemed necessary to adequately characterize smoking, abstinence, and relapse given the approximate 12-hour half-life of breath CO [40]. Frequent breath CO collection has been used in previous studies with reasonable compliance rates [41-43]. We felt this amount of CO collection was reasonable, particularly given the reduced burden in the rCO group associated with remote visits. The time-stamped CO submissions required the participant to take a video of themselves providing a breath CO sample and displaying the value on camera. All videos were verified by staff for accuracy. Evening reports were prompted at the end of the 12-hour time block every night (based on participant preference) and asked about missed cigarette entries not logged since the last evening report (past 24 hours). Participants were compensated for each EMA session, and evening report completed and could earn a total of US $210 in the wCO group or US $244 in the rCO group for perfect compliance (more in the rCO group given the added burden of CO monitoring).

#### Compensation

All compensation was provided in US dollars. Participants in both groups could earn the same amount of compensation for the completion of study procedures (US $404 possible). Participants in the wCO group could earn US $140 for completing in-person study visits, US $210 for EMA sessions, US $40 for quit attempt abstinence (confirmed at day 9), and US $14 for data use on their own mobile device or incentive for study device return (if applicable). Participants in the rCO group could earn US $80 for in-person study visits, US $244 for EMA session completion, US $40 for quit attempt abstinence (paid 2× per day on days 7-8), and US $40 for data use on their own mobile device or incentive for study device return. Compensation was provided to participants in cash. Participants in the wCO were compensated at each weekly study visit. Participants in the rCO group were compensated at the end of the study but were informed of their total amount earned throughout to enhance motivation.

### Ethics Approval

All study procedures were reviewed and approved by the institutional review board (PRO 00060290) at the Medical University of South Carolina. Written informed consent was obtained from all participants by trained research staff prior to any study procedures being completed. Consent could be withdrawn at any time during the study. The study team followed all procedures to protect participant privacy and confidentiality. All data were deidentified with a participant ID, and all protected health information were kept separate from research records in locked cabinets or password-protected databases and were only accessible to approved research staff. Details of study compensation are provided above.

### Measures

#### Screening or Baseline Assessments

Demographic and smoking history was collected along with past 30-day cigarette use, other nicotine use, alcohol, and other drugs via timeline follow-back procedures [44,45]. Nicotine dependence was assessed via the Modified Fagerstrom Tolerance Questionnaire [46], as well as readiness to quit smoking (10-point Likert scale with 1 being not ready and 10 being extremely ready). All non-EMA data were collected and managed using Research Electronic Data Capture [47].

#### Remote Monitoring Assessments

We used previously established EMA items here [22,26,33], including cigarette craving, affect (ie, happy, stressed, relaxed, and bored), social context (ie, who they were with and smoking cues), recent consumption behaviors (ie, eating and drinking), and time since last cigarette. Most questions were on a 5-point Likert scale that ranged from 1=not at all to 5=extremely. EMA items were included in event-based and random prompt EMA sessions (up to 4 per day). Evening report assessments included questions on the number of cigarettes smoked, other nicotine use, alcohol use, other drug use, and any use of NRT. Any missed cigarette entries during the day were reported in the evening report. Timeline follow-back procedures were performed at each weekly check-in (in-person or by phone) for any missed evening reports for both groups.

#### Biochemical Verification

Breath CO was captured twice per day (morning and evening) for the rCO group starting at day 1 and for the duration of the study or at weekly in-person study visits for the wCO group. Breath CO was captured using Bedfont Scientific Ltd breath CO monitors (piCO Smokerlyzer and the Micro^+^ basic monitors).

### Outcomes

#### Agreement (Primary Outcome)

Agreement between self-reported (EMA-based) past 24-hour smoking and CO-verified smoking was calculated (yes or no) at common time points (days 9, 14, 21, 28, and 35) for both groups.

#### Retention (Secondary Outcome)

Retention was defined as completing study visit procedures (in-person or phone visit) at the postquit attempt visit (day 9) and at the end of the study visit (day 35).

#### EMA Compliance (Secondary Outcome)

EMA session compliance for both groups was defined as the mean proportion of event-based and random prompt EMA sessions (calculated separately) and evening reports completed versus expected. Event-based EMA sessions relied on cigarette entries being logged and therefore, no expected number was known, and calculations were based on events reported.

#### Abstinence (Secondary Outcome)

Abstinence was defined based on negative breath CO samples and based on CO-confirmed self-reported abstinence at 2 time points. Abstinence was defined as a negative breath CO and self-reported abstinence from cigarettes during the quit attempt (days 7-8). For the rCO participants, abstinence was defined as having at least 3 out of 4 negative CO measures (≤6 ppm or a 75% decrease from mean prequit CO). For the wCO group, since there was only 1 time point available to determine abstinence, it was defined as a CO ≤6 ppm or a 75% decrease from prequit CO at the day 9 visit. End of study abstinence was defined as CO-confirmed self-reported 7-day point prevalence abstinence (PPA) at day 35. Abstinence is presented for CO confirmed only and CO-confirmed self-reported abstinence.

### Statistical Analyses

Standard descriptive statistics were used to summarize demographic and smoking characteristics for this study cohort as well as stratified by group (rCO vs wCO) and were compared across randomized groups using nonparametric tests. Within each study group, agreement between self-reported (EMA) and CO-verified smoking was estimated for each day using Cohen κ statistic [48]. Generalized estimating equations were used to assess group difference in agreement at common time points across both groups (days 9, 14, 21, 28, and 35). Using the logit function for binary responses, agreement (no vs yes) was regressed on group membership and study day (time-varying covariate). We selected correlation structures using quasi-information criterion minimization [49]. Models were fit using exchangeable correlation structures, and model-based means were extracted for day level between group estimates. Figure 2 also shows the number of participants in each group who were retained at study visits, and participants who had CO data available for analyses (note that CO was collected as part of study visits for the wCO group but through the app for the rCO group).

To examine group differences in retention, logistic regression with a sandwich variance estimate [50] was used, and risk ratios (RRs) with 95% CIs are presented. Group comparisons were conducted using a Wilcoxon Rank Sum test statistic. Abstinence was compared between groups using logistic regression with a sandwich variance estimate [50]. Analyses were intent-to-treat, and missing abstinence data were imputed (missing=smoking). All analyses were performed using SAS (version 9.4; SAS Institute Inc), and no adjustments were made to the presented *P* values.

This study was powered to detect a difference in the percent agreement of 24-hour self-reported and CO-verified abstinence between those randomized to the rCO versus the wCO group. Participants in both groups self-reported cigarettes smoked per day, but the rCO group had 58 possible CO samples during this study, while those in the wCO group had only 4 CO samples collected at in-person visits (Figure 2). As such, percent agreement between CO and self-report was assessed within and between groups at common time points. High agreement within the rCO group was expected due to the inclusion of remote biochemical verification (>93%). This study was powered to detect a percent agreement difference between groups of at least 13%, requiring 45 subjects per group (N=90 total) to detect a difference with a type I error rate of 5%.

## Results

Overall, the sample was majority male (n=22, 56%), White (n=29, 74%), and the average age was 21.6 (SD 2.1) years. Participants began smoking on average at 15 (SD 3.2) years old and reported 9.2 cigarettes per day (SD 5.1) at screening (Table 1).

**Table 1:** Demographics and smoking characteristics of the study sample (N=39), stratified by experimental group.

				Overall (N=39)		wCO^a^ (n=19)		rCO^b^ (n=20)
**Demographics**								
	Age (years), mean (SD)		21.6 (2.1)		21.7 (2.2)		21.6 (2.1)	
	Female, n (%)		17 (44)		8 (42)		9 (45)	
	**Race, n (%)**							
		White	29 (74)		13 (68)		16 (80)^c^	
		African American	5 (13)		5 (26)		0 (0)	
		More than 1 race or unknown	5 (13)		1 (5)		4 (20)	
		Hispanic or Latinx	6 (15)		3 (16)		3 (15)	
	**Education, n (%)**							
		High school or less	14 (36)		8 (42)		6 (30)	
		Some colleges or more	25 (64)		11 (58)		14 (70)	
	**Employment, n (%)**							
		Full time	18 (46)		10 (53)		8 (40)	
		Part time	12 (31)		5 (26)		7 (35)	
		Unemployed	9 (23)		4 (21)		5 (25)	
**Smoking characteristics, mean (SD)**								
	Age at smoking initiation (years)		15.3 (3.2)		16.2 (2.1)		14.5 (3.8)	
	Past quit attempts		3.4 (3.5)		2.9 (3.2)		3.9 (3.7)	
	Cigarettes per day (screening)		9.2 (5.1)		9.2 (4.9)		9.2 (5.4)	
	Breath CO (ppm; screening)		16.4 (10.5)		16.1 (10.7)		16.7 (10.6)	
	Modified Fagerstrom Tolerance Questionnaire		3.6 (1.6)		3.6 (1.3)		3.6 (1.8)	
	Readiness to quit (10-point scale)		7.8 (1.9)		7.6 (2.1)		8.0 (1.7)	

^a^wCO: weekly carbon monoxide.

^b^rCO: remote carbon monoxide.

^c^*P=*.04, calculated using Fisher exact test.

Agreement analyses were conducted using available self-reported smoking and CO (Table 2). When examining agreement between CO and self-reported past 24-hour smoking within the group, the wCO group exhibited moderate agreement for days 9, 14, 21, and 28 and poor agreement on day 35 (end of treatment). The rCO group exhibited moderate agreement on days 9 and 14, poor agreement on days 21 and 35, and perfect agreement on day 28 (with a limited sample of 5 participants). Agreement did not exceed 75% for either group at any time point, with the exception at day 28. Rates of missing data were higher in the rCO group (13%-64%) compared to the wCO group (11%-22%). Odds ratios between groups are presented in Table 2, and statistically significant differences were found for agreement. Using all time points, randomized treatment assignment was not significantly associated with agreement (odds ratio 0.82, 95% CI 0.35-1.92).

**Table 2:** Agreement between past 24-hour self-reported smoking and breath carbon monoxide (CO) at postquit attempt common time points. Missing data were not imputed in this analysis.

Study day	wCO^a^ (N=19 randomized)		rCO^b^ (N=20 randomized)		Odds ratio (95% CI)
	Agreement (yes), n/N (%)	Cohen κ	Agreement (yes), n/N (%)	Cohen κ	
9	11/16 (69)	0.23	8/11 (73)	0.46	1.09 (0.77-1.52)
14	12/16 (75)	0.20	7/11 (64)	0.21	0.90 (0.63-1.28)
21	12/16 (75)	0.26	6/11 (55)	0.04	0.80 (0.56-1.16)
28	9/14 (64)	0.22	5/5 (100)	1.00	N/A^c^
35	8/16 (50)	0.00	6/14 (43)	–0.04	0.93 (0.65-1.34)

^a^wCO: weekly carbon monoxide.

^b^rCO: remote carbon monoxide.

^c^N/A: not applicable.

Retention, compliance, and abstinence rates are shown in Table 3. Following the quit attempt (day 9), 37 of 39 (95%) participants remained actively enrolled (wCO=18/19, 95%; RR 1.0, 95% CI 0.86-1.16; rCO=19/20, 95% vs *P*=.98). At the final study visit (day 35), wCO group retention was 90% (17/19) compared to 75% (15/20) in the rCO group (RR 1.2, 95% CI 0.88-1.62; *P*=.26), but this was not statistically different.

**Table 3:** Study retention, remote session compliance, and smoking abstinence following the quit attempt (day 9) and at the end of treatment (day 35) for carbon monoxide (CO)–confirmed abstinence only and CO-confirmed self-reported point prevalence abstinence compared across groups^a^.

		Overall (N=39)	wCO^b^ (n=19)	rCO^c^ (n=20)	Risk ratio (95% CI)	*P* value
**Study retention, n (%)**						
	Day 9	37 (95)	18 (95)	19 (95)	1.0 (0.9 to 1.2)	.98
	Day 35	32 (82)	17 (90)	15 (75)	1.2 (0.9 to 1.6)	.26
**Remote session compliance, mean % (SD)**						
	Event-based (smoking) sessions	96 (16.6)	93 (23.1)	98 (6.7)	–5.1 (–16.0 to 5.8)	.24
	Random prompt sessions	47 (25.)	57 (25.8)	38 (21.1)	19.5 (4.2 to 34.7)	.01
	Evening reports	71 (28.1	74 (29.2)	68 (27.6)	5.8 (–12.6 to 24.2)	.36
**Day 9 abstinence, n/N (%)**						
	CO confirmed only	17/39 (44)	11/19 (58)	6/20 (30)	1.9 (0.9 to 4.3)	.10
	CO-confirmed 2-day SR^d^ abstinence	11/39 (28)	7/19 (37)	4/20 (20)	1.8 (0.6 to 5.4)	.28
**Day 35 (end of study) abstinence, n/N (%)**						
	CO confirmed only	10/39 (26)	8/19 (42)	2/20 (10)	4.21 (1.04 to 17.06)	.04
	CO-confirmed 7-day SR abstinence	2/39 (5)	2/19 (11)	0/20 (0)	—^e^	.23^f^

^a^Remote session compliance is noted as the mean (SD) percentage of EMA sessions and evening reports completed out of the number expected. EMA sessions include event-based (smoking) and random session prompts. Missing abstinence data were imputed as missing=smoking.

^b^wCO: weekly CO.

^c^rCO: remote CO.

^d^SR: self-reported.

^e^Not available.

^f^Calculated using Fisher exact test due to zero cell counts.

Sessions with predictable delivery times, including event-based EMA sessions (sessions immediately following a cigarette entry) and evening reports, showed high rates of completion: 96% (SD 16.8) for event-based EMA sessions and 71% (SD 28.1) for evening reports. Mean event-based EMA sessions were similarly high between the rCO and wCO groups: rCO=98% sessions completed (SD 6.7) versus wCO=93% sessions completed (SD 23.1); *P*=.24. Similarly, evening report compliance was consistent between groups: rCO=68% (SD 27.6) versus wCO=74% (SD 29.2); *P*=.36. Random prompt EMA sessions had lower rates of compliance, which were significantly lower in the rCO group as compared to the wCO group: rCO=38% (SD 21.1) versus wCO=57% (SD 25.8; *P*=.01).

Quit attempt (day 9) abstinence based on breath CO only found numerically lower rates of abstinence in the rCO group as compared to wCO group (rCO=6/20, 30% vs wCO=11/19, 58%; RR 1.95, 95% CI 0.89-4.26; *P*=.10). When assessing abstinence based on CO-confirmed 2-day PPA (self-reported abstinence verified by CO), results followed a similar pattern; not statistically different but numerically favored the wCO group (rCO=4/20, 20% vs wCO=7/19, 37%; RR 1.84, 95% CI 0.62-5.44; *P=*.28). End of study (day 35) abstinence determined by breath CO only showed that abstinence was significantly lower in the rCO group as compared to the wCO group (rCO=2/20, 10% vs wCO=8/19, 42%; RR 4.21, 95% CI 1.04-17.06; *P=*.04). Abstinence determined by CO-confirmed 7-day PPA (self-report verified by CO) showed a similar trend and was numerically lower in the rCO group as compared to the wCO group but not statistically significant, and only 2 wCO group participants were abstinent (rCO=0/20, 0% vs EMA=2/19, 11%). Between-group comparisons were conducted based on available CO and self-reported data, and results were similar to intent-to-treat results (not presented here).

## Discussion

### Overview of Findings

The inclusion of frequent remote breath CO capture added to EMA self-report [22] compared to EMA without rCO in young adults during a brief cessation study did not yield statistically different rates of agreement in self-reported smoking, compliance with EMA sessions, or retention. Though not statistically significant, rates of agreement, compliance, and retention were numerically higher in the wCO group. Smoking abstinence, confirmed through breath CO and self-reported smoking, was low, yet numerically higher in the wCO group at day 9 and statistically different at day 35, favoring the wCO group.

### Remote Biochemical Verification of Smoking

Agreement in past 24-hour self-reported smoking and breath CO was poor to moderate across both groups. It was hypothesized that rCO capture would lead to a higher agreement, which was not supported. Indeed, agreement was numerically higher (though not statistically significant) in the wCO group. A related finding was that CO-confirmed abstinence and CO-verified 7-day PPA for all participants revealed numerically lower abstinence in the rCO group compared to the wCO group following the quit attempt and statistically lower abstinence rates at the end of treatment (CO-confirmed abstinence, not 7-day PPA). Lower rates of abstinence in the rCO group cannot be explained by treatment differences or readiness to quit between groups, so it is likely that the accurate collection of abstinence through biochemical verification was the driver of group differences. These results suggest that more frequent CO assessment may provide a more accurate picture of abstinence, though it is also possible that the inclusion of rCO led to measurement reactivity, whereby the process of monitoring may have influenced smoking during the quit attempt and throughout monitoring. Research has shown evidence of measurement reactivity during EMA monitoring of smoking cessation [51], and it is possible that the addition of rCO monitoring in this study compounded this effect. Continual observation of CO-confirmed smoking during a cessation study may have even resulted in an abstinence violation effect [52] or the tendency to use following the violation of an abstinence goal, which could have led to increased smoking as a result of monitoring. rCO may have also been perceived as replacing the need to accurately report smoking since CO values indicate the presence of behavior. Further, motivation to continue frequent CO submissions and self-report may have been reduced after a lapse during the monitoring period. Remote biochemical verification of smoking may be a useful advancement to better detect *when* relapse has occurred to intervene rapidly, though a dynamic system that tailors the frequency of CO collection based on current smoking status (smoking, abstinence, and times of high risk) may function more effectively.

### Study Retention

Study retention following the quit attempt was high (ie, 95%), was not statistically different between groups, and was comparable to other studies using an EMA monitoring protocol leading up to a quit attempt [53,54]. Although not statistically significant, wCO group retention was numerically higher (90% vs 75%) than rCO group retention at the end of the study. This was unexpected given that the inclusion of technology to remotely capture CO was hypothesized to improve retention rates, as in-person visits were not regularly needed. This suggests potential value in the established relationship between staff and participants through in-person visits that may not be replaced through remote means. It is also possible that weekly in-person visits required for the wCO group made study participation more salient and increased commitment to completion. In addition, the added burden of frequent rCO collection and video upload through the app (2× per day for 5 weeks) may have adversely impacted retention. CO collection fatigue may have occurred, which is an important consideration for the inclusion of remote monitoring in future trials. Even so, results indicate that in the short term, the addition of remote biochemical verification to confirm self-reported smoking has the potential to provide fine-grained data during quit attempts without adversely affecting retention.

### Compliance With EMA Sessions

Finally, EMA compliance was consistent between groups for sessions with predictable delivery times (event-based sessions and evening reports) but not for random prompt sessions, which included a CO sample for the rCO group. Random prompt session compliance was significantly lower in the rCO group, which is likely a function of the increased burden of session completion (ie, time to record and upload the video). Sessions may have been prompted when participants did not have the CO device readily accessible, which may have resulted in skipping the session completely; therefore, missing the self-report data as well as CO collection. Random prompt sessions that require equipment to be available may adversely affect compliance. Particularly for CO, which can detect smoking for 12-24 hours, participant-initiated CO samples may be preferable and yield better compliance rates. Despite the difference in compliance between groups, overall compliance across session types (ie, 71%) was comparable to pooled compliance rates (ie, 75%) found in a recent meta-analysis of EMA studies [55].

### Limitations

This study had several limitations. First, missing data were prevalent, particularly in the rCO group, which affected the ability to detect group differences. This may have been the result of burden associated with logging each cigarette smoked per day for 5 weeks and completing random sessions throughout that time and insufficient compensation to promote continued engagement. Further, because this group was required to provide a CO sample twice per day during the study, the data may not be missing at random, but rather be due to the additional burden to frequent sampling. Second, while all participants were interested in quitting smoking (5 on a 10-point Likert scale to be included) and received treatment (NRT, brief counseling, and incentives), treatment was minimal and may not have been sufficient to promote continued abstinence and sustained motivation throughout the trial. Treatment-seeking participants may be more likely to engage in monitoring activities, particularly if cessation content is provided through the same monitoring app. Third, we were underpowered to detect differences in agreement between self-reported smoking and CO measurement. Due to a slow rate of enrollment and the increased prevalence of nicotine vaping during study recruitment, our proposed sample size was not successfully met. Numeric trends favoring the wCO group on many outcomes were found, which indicates that even with the proposed sample size, our hypotheses may not have been supported. Fourth, while study procedures across experimental conditions were kept similar, they varied between remote versus in-person depending on the condition, which may have contributed to group differences. This difference may have even played a larger role in group differences beyond rCO collection. Finally, those participants with serious or unstable psychiatric disorders were excluded from participating, which limits the generalizability of the study findings. Participants with psychiatric disorders who were deemed stable (ie, stable dose of medication and seeing a provider regularly) were assessed and included, though this highlights the need to focus young adult smoking research on those with co-occurring disorders in order accurately represent the population in research studies.

### Conclusions

The goal of this study was to evaluate the benefits of technological advancement for young adult smoking cessation research methods. The rCO group did not show benefit over the in-person group (wCO), and in some cases, outcomes were worse for the rCO group. Remote methods did not translate into improved compliance or retention, though they may provide a more accurate and fine-grained characterization of the quit, lapse, and relapse processes. EMA is an important tool in this regard, as assessment in one’s natural environment maximizes ecological validity [56], yet common EMA methods may suffer other threats to validity, such as intentional or unintentional biases in responding. Using objective measures, such as remote biochemical verification in conjunction with EMA, may be an important way forward in dynamically assessing smoking relapse [22,37]. However, others have warned against the unnecessary inclusion of remote biochemical verification in smoking studies as standard practice when not sufficiently warranted [57]. Results from this study help to inform that debate and suggest that technological enhancement may improve data accuracy through objective measurement but may also introduce compliance barriers that could result in higher rates of missing data. The inclusion of technology to inform smoking cessation research and intervention delivery among young adults should consider (1) the research question and necessity of biochemical verification and then (2) how to seamlessly incorporate monitoring into personalized and dynamic systems to avoid the added burden and detrimental effects to compliance and honesty in self-report to not compromise the goals of this study.

## Abbreviations

CO: carbon monoxide
EMA: ecological momentary assessment
NRT: nicotine replacement therapy
PPA: point prevalence abstinence
ppm: parts per million
rCO: remote carbon monoxide
RR: risk ratio
wCO: weekly carbon monoxide

## Data Availability

The final data set supporting this paper will be made available to qualified requestors following the publication of main study findings and the removal of all human subject identifying information in accordance with the National Institutes of Health Data Sharing Policies. Requests for deidentified data access should be made directly to the corresponding author (EAM).
